# 5-Nitro-1-(prop-2-yn-1-yl)-2,3-dihydro-1*H*-1,3-benzodiazol-2-one

**DOI:** 10.1107/S1600536812013177

**Published:** 2012-03-31

**Authors:** Younes Ouzidan, Youssef Kandri Rodi, Hafid Zouihri, El Mokhtar Essassi, Seik Weng Ng

**Affiliations:** a Laboratoire de Chimie Organique Appliquée, Pôle de Compétences Pharmacochimie, Université Mohammed V-Agdal, BP 1014 Avenue Ibn Batout, Rabat, Morocco; b Laboratoire de Chimie Organique Appliquée, Faculté des Sciences et Techniques, Université Sidi Mohamed Ben Abdallah, Fés, Morocco; c CNRST Division UATRS, Angle Allal Fassi/FAR, BP 8027 Hay Riad, Rabat, Morocco; d Laboratoire de Chimie Organique Hétérocyclique, Pôle de Compétences Pharmacochimie, Université Mohammed V-Agdal, BP 1014 Avenue Ibn Batout, Rabat, Morocco; e Department of Chemistry, University of Malaya, 50603 Kuala Lumpur, Malaysia; f Chemistry Department, King Abdulaziz University, PO Box 80203 Jeddah, Saudi Arabia

## Abstract

In the two independent mol­ecules of the title compound, C_10_H_7_N_3_O_3_, the nitro substitutent is twisted slightly with respect to the benzodiazol fused-ring system [dihedral angles = 4.9 (3) and 8.5 (1)°]. The two independent mol­ecules are disposed about a pseudo inversion center and are held together by N—H⋯O hydrogen bonds. The supramolecular dimer is essentially planar [dihedral angle between the fused rings = 2.0 (1)°]. Adjacent dimers are linked by acetyl­ene–nitro C—H⋯O inter­actions, generating a ribbon motif along (110).

## Related literature

For related structures, see: Ouzidan *et al.* (2011*a* ▶,*b* ▶,*c* ▶).
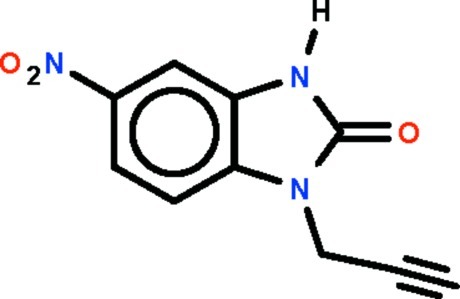


## Experimental

### 

#### Crystal data

C_10_H_7_N_3_O_3_
*M* *_r_* = 217.19
Triclinic, 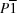

*a* = 7.2541 (2) Å
*b* = 10.0362 (2) Å
*c* = 14.6793 (3) Å
α = 100.978 (1)°
β = 92.047 (1)°
γ = 109.043 (1)°
*V* = 986.20 (4) Å^3^
*Z* = 4
Mo *K*α radiation
μ = 0.11 mm^−1^
*T* = 293 K
0.27 × 0.22 × 0.13 mm

#### Data collection

Bruker APEX DUO diffractometer
20686 measured reflections
4506 independent reflections
2855 reflections with *I* > 2σ(*I*)
*R*_int_ = 0.032

#### Refinement

*R*[*F* ^2^ > 2σ(*F* ^2^)] = 0.045
*wR*(*F* ^2^) = 0.158
*S* = 1.05
4506 reflections
305 parameters
H atoms treated by a mixture of independent and constrained refinement
Δρ_max_ = 0.20 e Å^−3^
Δρ_min_ = −0.24 e Å^−3^

### 

Data collection: *APEX2* (Bruker, 2010 ▶); cell refinement: *SAINT* (Bruker, 2010 ▶); data reduction: *SAINT*; program(s) used to solve structure: *SHELXS97* (Sheldrick, 2008 ▶); program(s) used to refine structure: *SHELXL97* (Sheldrick, 2008 ▶); molecular graphics: *X-SEED* (Barbour, 2001 ▶); software used to prepare material for publication: *publCIF* (Westrip, 2010 ▶).

## Supplementary Material

Crystal structure: contains datablock(s) global, I. DOI: 10.1107/S1600536812013177/bt5859sup1.cif

Structure factors: contains datablock(s) I. DOI: 10.1107/S1600536812013177/bt5859Isup2.hkl

Supplementary material file. DOI: 10.1107/S1600536812013177/bt5859Isup3.cml

Additional supplementary materials:  crystallographic information; 3D view; checkCIF report

## Figures and Tables

**Table 1:** Hydrogen-bond geometry (Å, °)

*D*—H⋯*A*	*D*—H	H⋯*A*	*D*⋯*A*	*D*—H⋯*A*
N1—H1⋯O4	0.96 (2)	1.81 (3)	2.766 (2)	173 (2)
N4—H4⋯O1	0.92 (2)	1.91 (2)	2.823 (2)	172 (2)
C4—H41⋯O3^i^	0.92 (4)	2.39 (4)	3.231 (4)	153 (3)
C14—H141⋯O6^ii^	0.88 (3)	2.51 (3)	3.383 (4)	169 (3)

Symmetry codes: (i) 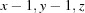; (ii) 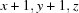.

**Fig. 1.**
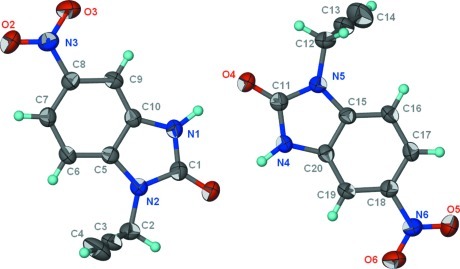
Anisotropic displacement ellipsoid plot (Barbour, 2001) of the two independent molecules of C10H7N3O3 molecule at the 50% probability level; hydrogen atoms are drawn as spheres of arbitrary radius.

**Fig. 2.**
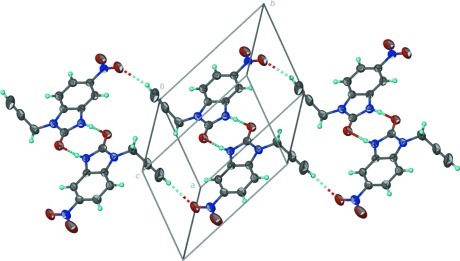
Anisotropic displacement ellipsoid plot (Barbour, 2001) of the hydrogen-bonded ribbon motif.

## supplementary crystallographic information

### Comment

Benzodiazoles are of interest owing to their pharmacological properties. When the parent compound, benzodiazol-2-one, reacts with propargyl bromide, both amino groups are alkylated to give 1,3-bis(prop-2-ynyl)-benziodiazol-2-one (Ouzidan *et al.*, 2011*b*). The presence of an electron-withdrawing nitro group allows only one amino group to be alklylated, as noted from the reactions of 5-nitrobenzodiazol-2-onezol with *n*-octyl bromide and *n*-nonyl bromide (Ouzidan *et al.*, 2011*b*, 2011*c*). In the two independent molecules of C_10_H_7_N_3_O_3 _(Scheme I), the nitro substitutent is slightly bent with respect to the benzodiazol fused-ring (Fig. 2). The two are disposed about a false inversion center, and are held together by N–H···O hydrogen bonds. Adjacent dimers are linked by C–H_acetylene_···O_nitro_ interactions to generate a ribbon motif (Fig. 2).

### Experimental

To a mixture of 5-nitro-1*H*-benzodiazol-2(3*H*)-one (0.25 g, 1.5 mmol), potassium carbonate (0.35 g, 2.5 mmol), tetra-*n*-butylammonium bromide (0.1 g,0.2 mmol) in DMF (15 ml) was added propargyl bromide (0.14 ml, 1.6 mmol). Stirring was continued at room temperature for 6 hours. The salt was removed by filtration and the filtrate concentrated under reduced pressure. The residue was separated by chromatography on a column of silica gel with ethylacetate/hexane; yellow crystals were obtained upon evaporation of the solvent.

### Refinement

All H atoms were located in a difference map. The aromatic and methylene H-atoms were placed in calculated positions (C–H 0.93–0.97 Å) and were included in the refinement in the riding model approximation, with *U*(H) set to 1.2*U*(C).

The amino and acetylenic H-atoms were freely refined.

The (0 0 1) reflection was omitted owing to bad disagreement.

### Crystal data

**Table:**

C_10_H_7_N_3_O_3_	*Z* = 4
*M**_r_* = 217.19	*F*(000) = 448
Triclinic, *P*1	*D*_x_ = 1.463 Mg m^−^^3^
Hall symbol: -P 1	Mo *K*α radiation, λ = 0.71073 Å
*a* = 7.2541 (2) Å	Cell parameters from 5674 reflections
*b* = 10.0362 (2) Å	θ = 2.2–28.0°
*c* = 14.6793 (3) Å	µ = 0.11 mm^−^^1^
α = 100.978 (1)°	*T* = 293 K
β = 92.047 (1)°	Prism, yellow
γ = 109.043 (1)°	0.27 × 0.22 × 0.13 mm
*V* = 986.20 (4) Å^3^	

### Data collection

**Table:**

Bruker APEX DUO diffractometer	2855 reflections with *I* > 2σ(*I*)
Radiation source: fine-focus sealed tube	*R*_int_ = 0.032
Graphite monochromator	θ_max_ = 27.5°, θ_min_ = 2.2°
ω scans	*h* = −9→9
20686 measured reflections	*k* = −13→12
4506 independent reflections	*l* = −19→19

### Refinement

**Table:**

Refinement on *F*^2^	Primary atom site location: structure-invariant direct methods
Least-squares matrix: full	Secondary atom site location: difference Fourier map
*R*[*F*^2^ > 2σ(*F*^2^)] = 0.045	Hydrogen site location: inferred from neighbouring sites
*wR*(*F*^2^) = 0.158	H atoms treated by a mixture of independent and constrained refinement
*S* = 1.05	*w* = 1/[σ^2^(*F*_o_^2^) + (0.0746*P*)^2^ + 0.2589*P*] where *P* = (*F*_o_^2^ + 2*F*_c_^2^)/3
4506 reflections	(Δ/σ)_max_ = 0.001
305 parameters	Δρ_max_ = 0.20 e Å^−^^3^
0 restraints	Δρ_min_ = −0.24 e Å^−^^3^

### Fractional atomic coordinates and isotropic or equivalent isotropic displacement parameters (Å^2^)

**Table:**

	*x*	*y*	*z*	*U*_iso_*/*U*_eq_	
O1	0.4863 (2)	0.28214 (16)	0.32061 (10)	0.0583 (4)	
O2	0.2729 (3)	0.70175 (19)	−0.06407 (12)	0.0780 (5)	
O3	0.4106 (3)	0.84006 (18)	0.06741 (13)	0.0826 (6)	
O4	0.7053 (2)	0.67457 (15)	0.41638 (10)	0.0556 (4)	
O5	1.0136 (4)	0.2778 (2)	0.79280 (13)	0.1056 (8)	
O6	0.8109 (3)	0.13338 (17)	0.67890 (13)	0.0826 (6)	
N1	0.4993 (2)	0.47954 (18)	0.25677 (11)	0.0459 (4)	
N2	0.3361 (2)	0.26206 (16)	0.17380 (10)	0.0438 (4)	
N3	0.3367 (2)	0.72031 (19)	0.01736 (13)	0.0535 (4)	
N4	0.7143 (2)	0.48027 (17)	0.47997 (11)	0.0416 (4)	
N5	0.8738 (2)	0.70106 (16)	0.55973 (10)	0.0429 (4)	
N6	0.9101 (3)	0.25456 (19)	0.72058 (13)	0.0583 (5)	
C1	0.4468 (3)	0.3367 (2)	0.25799 (13)	0.0447 (4)	
C2	0.2539 (3)	0.1058 (2)	0.14690 (15)	0.0555 (5)	
H2A	0.3007	0.0644	0.1936	0.067*	
H2B	0.2988	0.0734	0.0880	0.067*	
C3	0.0406 (4)	0.0549 (2)	0.13742 (15)	0.0585 (6)	
C4	−0.1296 (5)	0.0194 (3)	0.1295 (2)	0.0859 (9)	
C5	0.3154 (3)	0.35855 (19)	0.12192 (12)	0.0391 (4)	
C6	0.2169 (3)	0.3381 (2)	0.03563 (13)	0.0450 (4)	
H6	0.1471	0.2461	0.0011	0.054*	
C7	0.2259 (3)	0.4598 (2)	0.00241 (13)	0.0454 (4)	
H7	0.1628	0.4505	−0.0559	0.054*	
C8	0.3287 (3)	0.5950 (2)	0.05579 (13)	0.0418 (4)	
C9	0.4281 (3)	0.6184 (2)	0.14315 (13)	0.0428 (4)	
H9	0.4961	0.7107	0.1778	0.051*	
C10	0.4197 (2)	0.49734 (19)	0.17501 (12)	0.0391 (4)	
C11	0.7577 (3)	0.6231 (2)	0.47796 (13)	0.0432 (4)	
C12	0.9419 (3)	0.8575 (2)	0.58554 (15)	0.0552 (5)	
H12A	0.8899	0.8870	0.6430	0.066*	
H12B	0.8916	0.8943	0.5372	0.066*	
C13	1.1548 (4)	0.9205 (2)	0.59851 (15)	0.0621 (6)	
C14	1.3251 (5)	0.9698 (3)	0.6118 (2)	0.0942 (10)	
C15	0.9034 (3)	0.60770 (18)	0.61264 (12)	0.0381 (4)	
C16	1.0082 (3)	0.6333 (2)	0.69790 (13)	0.0452 (4)	
H16	1.0756	0.7267	0.7310	0.054*	
C17	1.0092 (3)	0.5145 (2)	0.73252 (13)	0.0463 (5)	
H17	1.0786	0.5271	0.7898	0.056*	
C18	0.9065 (3)	0.3768 (2)	0.68142 (13)	0.0432 (4)	
C19	0.7999 (3)	0.34865 (19)	0.59536 (12)	0.0404 (4)	
H19	0.7318	0.2552	0.5625	0.048*	
C20	0.8012 (2)	0.46764 (19)	0.56177 (12)	0.0371 (4)	
H1	0.574 (3)	0.554 (3)	0.3093 (17)	0.073 (7)*	
H4	0.645 (3)	0.409 (2)	0.4297 (16)	0.060 (6)*	
H41	−0.263 (6)	−0.002 (4)	0.122 (3)	0.138 (14)*	
H141	1.454 (5)	1.011 (3)	0.621 (2)	0.118 (12)*	

### Atomic displacement parameters (Å^2^)

**Table:**

	*U*^11^	*U*^22^	*U*^33^	*U*^12^	*U*^13^	*U*^23^
O1	0.0683 (9)	0.0531 (9)	0.0505 (8)	0.0149 (7)	−0.0098 (7)	0.0175 (7)
O2	0.0971 (13)	0.0713 (11)	0.0680 (11)	0.0265 (10)	−0.0142 (9)	0.0289 (9)
O3	0.1019 (14)	0.0448 (10)	0.0931 (13)	0.0164 (9)	−0.0181 (10)	0.0160 (9)
O4	0.0672 (9)	0.0501 (8)	0.0493 (8)	0.0188 (7)	−0.0090 (7)	0.0151 (7)
O5	0.169 (2)	0.0641 (12)	0.0750 (12)	0.0367 (12)	−0.0557 (13)	0.0130 (10)
O6	0.1049 (14)	0.0406 (9)	0.0919 (13)	0.0138 (9)	−0.0253 (10)	0.0156 (9)
N1	0.0484 (9)	0.0429 (9)	0.0398 (9)	0.0081 (7)	−0.0054 (7)	0.0082 (7)
N2	0.0512 (9)	0.0392 (9)	0.0398 (8)	0.0157 (7)	0.0000 (7)	0.0060 (7)
N3	0.0492 (10)	0.0468 (10)	0.0649 (12)	0.0145 (8)	0.0006 (8)	0.0172 (9)
N4	0.0445 (9)	0.0383 (9)	0.0376 (8)	0.0107 (7)	−0.0035 (7)	0.0050 (7)
N5	0.0526 (9)	0.0351 (8)	0.0396 (8)	0.0146 (7)	0.0018 (7)	0.0055 (7)
N6	0.0762 (12)	0.0428 (10)	0.0556 (11)	0.0211 (9)	−0.0062 (9)	0.0104 (8)
C1	0.0451 (10)	0.0465 (11)	0.0416 (10)	0.0147 (8)	0.0014 (8)	0.0092 (9)
C2	0.0770 (15)	0.0389 (11)	0.0518 (12)	0.0243 (10)	0.0004 (10)	0.0051 (9)
C3	0.0795 (16)	0.0329 (10)	0.0503 (12)	0.0084 (10)	−0.0016 (11)	−0.0006 (9)
C4	0.082 (2)	0.0561 (16)	0.088 (2)	−0.0025 (14)	0.0001 (16)	−0.0130 (14)
C5	0.0418 (9)	0.0385 (10)	0.0380 (9)	0.0159 (8)	0.0051 (7)	0.0064 (8)
C6	0.0519 (11)	0.0399 (10)	0.0384 (10)	0.0142 (8)	−0.0013 (8)	0.0008 (8)
C7	0.0472 (10)	0.0484 (11)	0.0404 (10)	0.0175 (9)	−0.0019 (8)	0.0081 (8)
C8	0.0400 (9)	0.0437 (10)	0.0446 (10)	0.0156 (8)	0.0057 (8)	0.0131 (8)
C9	0.0402 (10)	0.0371 (10)	0.0460 (11)	0.0087 (7)	0.0039 (8)	0.0046 (8)
C10	0.0356 (9)	0.0422 (10)	0.0363 (9)	0.0106 (7)	0.0031 (7)	0.0059 (8)
C11	0.0428 (10)	0.0440 (11)	0.0422 (10)	0.0144 (8)	0.0033 (8)	0.0089 (8)
C12	0.0725 (14)	0.0361 (11)	0.0553 (12)	0.0177 (10)	0.0026 (10)	0.0076 (9)
C13	0.0790 (17)	0.0394 (11)	0.0541 (13)	0.0046 (11)	−0.0032 (11)	0.0067 (10)
C14	0.081 (2)	0.0721 (19)	0.096 (2)	−0.0106 (16)	−0.0107 (17)	0.0092 (16)
C15	0.0429 (9)	0.0350 (9)	0.0360 (9)	0.0137 (7)	0.0070 (7)	0.0054 (7)
C16	0.0537 (11)	0.0372 (10)	0.0379 (10)	0.0119 (8)	−0.0001 (8)	−0.0008 (8)
C17	0.0558 (11)	0.0465 (11)	0.0344 (9)	0.0180 (9)	−0.0018 (8)	0.0032 (8)
C18	0.0498 (10)	0.0393 (10)	0.0415 (10)	0.0173 (8)	0.0039 (8)	0.0075 (8)
C19	0.0432 (10)	0.0337 (9)	0.0401 (10)	0.0111 (7)	0.0020 (8)	0.0021 (7)
C20	0.0365 (9)	0.0399 (10)	0.0337 (9)	0.0138 (7)	0.0040 (7)	0.0036 (7)

### Geometric parameters (Å, º)

**Table:**

O1—C1	1.226 (2)	C4—H41	0.92 (4)
O2—N3	1.224 (2)	C5—C6	1.377 (2)
O3—N3	1.219 (2)	C5—C10	1.406 (2)
O4—C11	1.229 (2)	C6—C7	1.382 (3)
O5—N6	1.215 (2)	C6—H6	0.9300
O6—N6	1.219 (2)	C7—C8	1.380 (3)
N1—C1	1.362 (2)	C7—H7	0.9300
N1—C10	1.380 (2)	C8—C9	1.389 (3)
N1—H1	0.96 (2)	C9—C10	1.367 (3)
N2—C5	1.381 (2)	C9—H9	0.9300
N2—C1	1.385 (2)	C12—C13	1.455 (3)
N2—C2	1.452 (2)	C12—H12A	0.9700
N3—C8	1.459 (2)	C12—H12B	0.9700
N4—C11	1.370 (2)	C13—C14	1.164 (4)
N4—C20	1.383 (2)	C14—H141	0.88 (3)
N4—H4	0.92 (2)	C15—C16	1.375 (2)
N5—C11	1.379 (2)	C15—C20	1.401 (2)
N5—C15	1.387 (2)	C16—C17	1.384 (3)
N5—C12	1.452 (2)	C16—H16	0.9300
N6—C18	1.457 (2)	C17—C18	1.387 (3)
C2—C3	1.455 (3)	C17—H17	0.9300
C2—H2A	0.9700	C18—C19	1.388 (3)
C2—H2B	0.9700	C19—C20	1.373 (2)
C3—C4	1.162 (4)	C19—H19	0.9300
			
C1—N1—C10	110.23 (16)	C7—C8—C9	123.72 (17)
C1—N1—H1	121.9 (14)	C7—C8—N3	118.08 (17)
C10—N1—H1	127.7 (14)	C9—C8—N3	118.18 (17)
C5—N2—C1	109.67 (15)	C10—C9—C8	115.85 (17)
C5—N2—C2	126.40 (16)	C10—C9—H9	122.1
C1—N2—C2	123.92 (16)	C8—C9—H9	122.1
O3—N3—O2	122.50 (18)	C9—C10—N1	131.69 (17)
O3—N3—C8	118.46 (18)	C9—C10—C5	121.39 (16)
O2—N3—C8	119.03 (18)	N1—C10—C5	106.92 (16)
C11—N4—C20	109.80 (15)	O4—C11—N4	127.80 (18)
C11—N4—H4	120.7 (13)	O4—C11—N5	125.54 (18)
C20—N4—H4	129.3 (14)	N4—C11—N5	106.66 (16)
C11—N5—C15	109.80 (15)	N5—C12—C13	112.49 (17)
C11—N5—C12	123.13 (16)	N5—C12—H12A	109.1
C15—N5—C12	126.98 (16)	C13—C12—H12A	109.1
O5—N6—O6	122.84 (18)	N5—C12—H12B	109.1
O5—N6—C18	118.59 (18)	C13—C12—H12B	109.1
O6—N6—C18	118.57 (17)	H12A—C12—H12B	107.8
O1—C1—N1	127.84 (18)	C14—C13—C12	177.7 (3)
O1—C1—N2	125.59 (18)	C13—C14—H141	177 (2)
N1—C1—N2	106.57 (16)	C16—C15—N5	131.48 (17)
N2—C2—C3	111.72 (17)	C16—C15—C20	122.06 (16)
N2—C2—H2A	109.3	N5—C15—C20	106.46 (15)
C3—C2—H2A	109.3	C15—C16—C17	117.40 (17)
N2—C2—H2B	109.3	C15—C16—H16	121.3
C3—C2—H2B	109.3	C17—C16—H16	121.3
H2A—C2—H2B	107.9	C16—C17—C18	119.75 (17)
C4—C3—C2	177.4 (3)	C16—C17—H17	120.1
C3—C4—H41	176 (2)	C18—C17—H17	120.1
C6—C5—N2	131.73 (17)	C19—C18—C17	123.75 (17)
C6—C5—C10	121.69 (17)	C19—C18—N6	118.16 (17)
N2—C5—C10	106.58 (15)	C17—C18—N6	118.09 (17)
C5—C6—C7	117.37 (17)	C20—C19—C18	115.76 (16)
C5—C6—H6	121.3	C20—C19—H19	122.1
C7—C6—H6	121.3	C18—C19—H19	122.1
C8—C7—C6	119.97 (17)	C19—C20—N4	131.45 (16)
C8—C7—H7	120.0	C19—C20—C15	121.28 (16)
C6—C7—H7	120.0	N4—C20—C15	107.28 (15)
			
C10—N1—C1—O1	−177.65 (19)	C20—N4—C11—O4	−179.86 (18)
C10—N1—C1—N2	1.7 (2)	C20—N4—C11—N5	0.1 (2)
C5—N2—C1—O1	177.68 (18)	C15—N5—C11—O4	−179.94 (18)
C2—N2—C1—O1	−1.5 (3)	C12—N5—C11—O4	3.2 (3)
C5—N2—C1—N1	−1.7 (2)	C15—N5—C11—N4	0.1 (2)
C2—N2—C1—N1	179.04 (16)	C12—N5—C11—N4	−176.74 (17)
C5—N2—C2—C3	−64.0 (2)	C11—N5—C12—C13	−120.6 (2)
C1—N2—C2—C3	115.0 (2)	C15—N5—C12—C13	63.0 (3)
C1—N2—C5—C6	−178.67 (19)	C11—N5—C15—C16	179.53 (19)
C2—N2—C5—C6	0.5 (3)	C12—N5—C15—C16	−3.7 (3)
C1—N2—C5—C10	1.1 (2)	C11—N5—C15—C20	−0.27 (19)
C2—N2—C5—C10	−179.73 (17)	C12—N5—C15—C20	176.45 (17)
N2—C5—C6—C7	−179.67 (18)	N5—C15—C16—C17	−179.89 (18)
C10—C5—C6—C7	0.6 (3)	C20—C15—C16—C17	−0.1 (3)
C5—C6—C7—C8	−0.8 (3)	C15—C16—C17—C18	−0.2 (3)
C6—C7—C8—C9	0.4 (3)	C16—C17—C18—C19	0.1 (3)
C6—C7—C8—N3	179.11 (16)	C16—C17—C18—N6	−179.55 (17)
O3—N3—C8—C7	172.97 (18)	O5—N6—C18—C19	175.1 (2)
O2—N3—C8—C7	−8.4 (3)	O6—N6—C18—C19	−4.0 (3)
O3—N3—C8—C9	−8.3 (3)	O5—N6—C18—C17	−5.2 (3)
O2—N3—C8—C9	170.30 (18)	O6—N6—C18—C17	175.7 (2)
C7—C8—C9—C10	0.1 (3)	C17—C18—C19—C20	0.2 (3)
N3—C8—C9—C10	−178.55 (15)	N6—C18—C19—C20	179.91 (16)
C8—C9—C10—N1	179.90 (18)	C18—C19—C20—N4	179.50 (18)
C8—C9—C10—C5	−0.3 (2)	C18—C19—C20—C15	−0.5 (2)
C1—N1—C10—C9	178.72 (19)	C11—N4—C20—C19	179.75 (18)
C1—N1—C10—C5	−1.1 (2)	C11—N4—C20—C15	−0.23 (19)
C6—C5—C10—C9	−0.1 (3)	C16—C15—C20—C19	0.5 (3)
N2—C5—C10—C9	−179.84 (15)	N5—C15—C20—C19	−179.68 (15)
C6—C5—C10—N1	179.78 (16)	C16—C15—C20—N4	−179.52 (16)
N2—C5—C10—N1	0.01 (19)	N5—C15—C20—N4	0.30 (18)

### Hydrogen-bond geometry (Å, º)

**Table:**

*D*—H···*A*	*D*—H	H···*A*	*D*···*A*	*D*—H···*A*
N1—H1···O4	0.96 (2)	1.81 (3)	2.766 (2)	173 (2)
N4—H4···O1	0.92 (2)	1.91 (2)	2.823 (2)	172 (2)
C4—H41···O3^i^	0.92 (4)	2.39 (4)	3.231 (4)	153 (3)
C14—H141···O6^ii^	0.88 (3)	2.51 (3)	3.383 (4)	169 (3)

Symmetry codes: (i) *x*−1, *y*−1, *z*; (ii) *x*+1, *y*+1, *z*.
